# Supplementary material for: A Novel Heterozygous Deletion Variant in KLOTHO Gene Leading to Haploinsufficiency and Impairment of Fibroblast Growth Factor 23 Signaling Pathway
Source: J Clin Med. 2019 Apr 12;8(4):500. doi: 10.3390/jcm8040500 (PMC6517886; doi:10.3390/jcm8040500)
Supplement: Supplementary file 1 [file jcm-08-00500-s001.zip › supplementary/captions.docx]

**Table S1.** Data of the two groups of hemodialysis patients with normal and elevated serum phosphate.

**File S1**. **Sequence data files of *KL* gene variant identified**. (**A**) KLOTHO_B1120_exon2_1F.ab1: sequence data of *KL* gene exon 2. (**B**) KLOTHO_B1120_cDNA_R-reverse: sequence data of *KL* cDNA.

**File S2. *EGR1* gene expression in HEK293 cells results assessed by quantitative RT-PCR.** EGR 1_gene expression analysis results.xls.

**Figure S1. Immunoblot of total and phosphorylated ERK1/2 in HEK293 cells at different time expositions.** HEK293 cells were transfected with empty vector (Control), full-length (FL) or p.Ile348Phefs*28 *KL* gene variant and treated with vehicle (PBS with BSA 0.1%; -) or recombinant FGF23 (100 ng/mL; +) for 60 min. Two replicates (#1 and #2) of the same experiment are shown.
