# Supplementary figures and images for: A Novel Heterozygous Deletion Variant in KLOTHO Gene Leading to Haploinsufficiency and Impairment of Fibroblast Growth Factor 23 Signaling Pathway
Source: J Clin Med. 2019 Apr 12;8(4):500. doi: 10.3390/jcm8040500 (PMC6517886; doi:10.3390/jcm8040500)

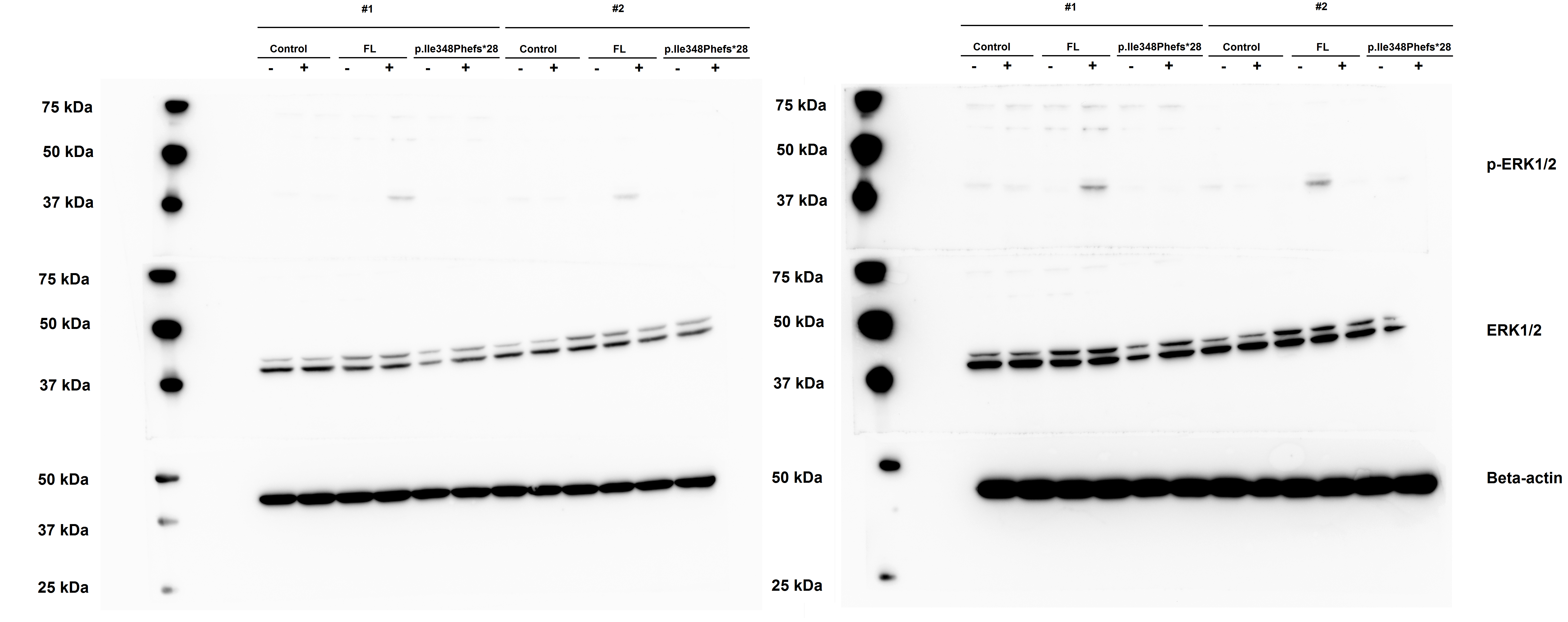

Supplement: Supplementary file 1 [file jcm-08-00500-s001.zip › supplementary/Figure S1_western blot antiERK1-2.tif]
